# Supplementary material for: Depth of the Steroid Core Location Determines the Mode of Na,K-ATPase Inhibition by Cardiotonic Steroids
Source: Int J Mol Sci. 2021 Dec 9;22(24):13268. doi: 10.3390/ijms222413268 (PMC8708600; doi:10.3390/ijms222413268)
Supplement: Supplementary file 1 [file ijms-22-13268-s001.zip › ijms-1467721-supplementary.pdf]

SUPPLEMENTARY MATERIALS

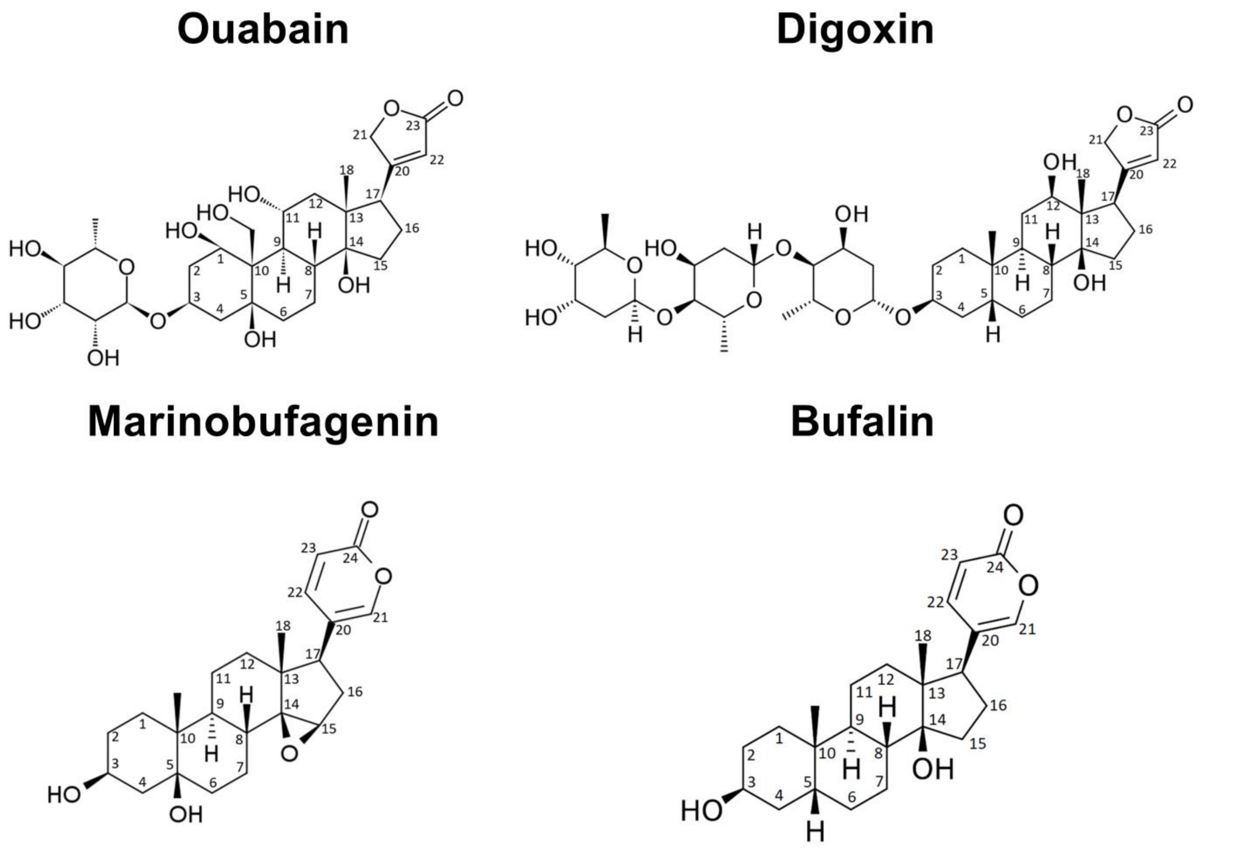

Figure S1. Structures of cardiotonic steroids. Cardenolides — ouabain and digoxin — are shown. Bufadienolides — marinobufagenin and bufalin — are shown.

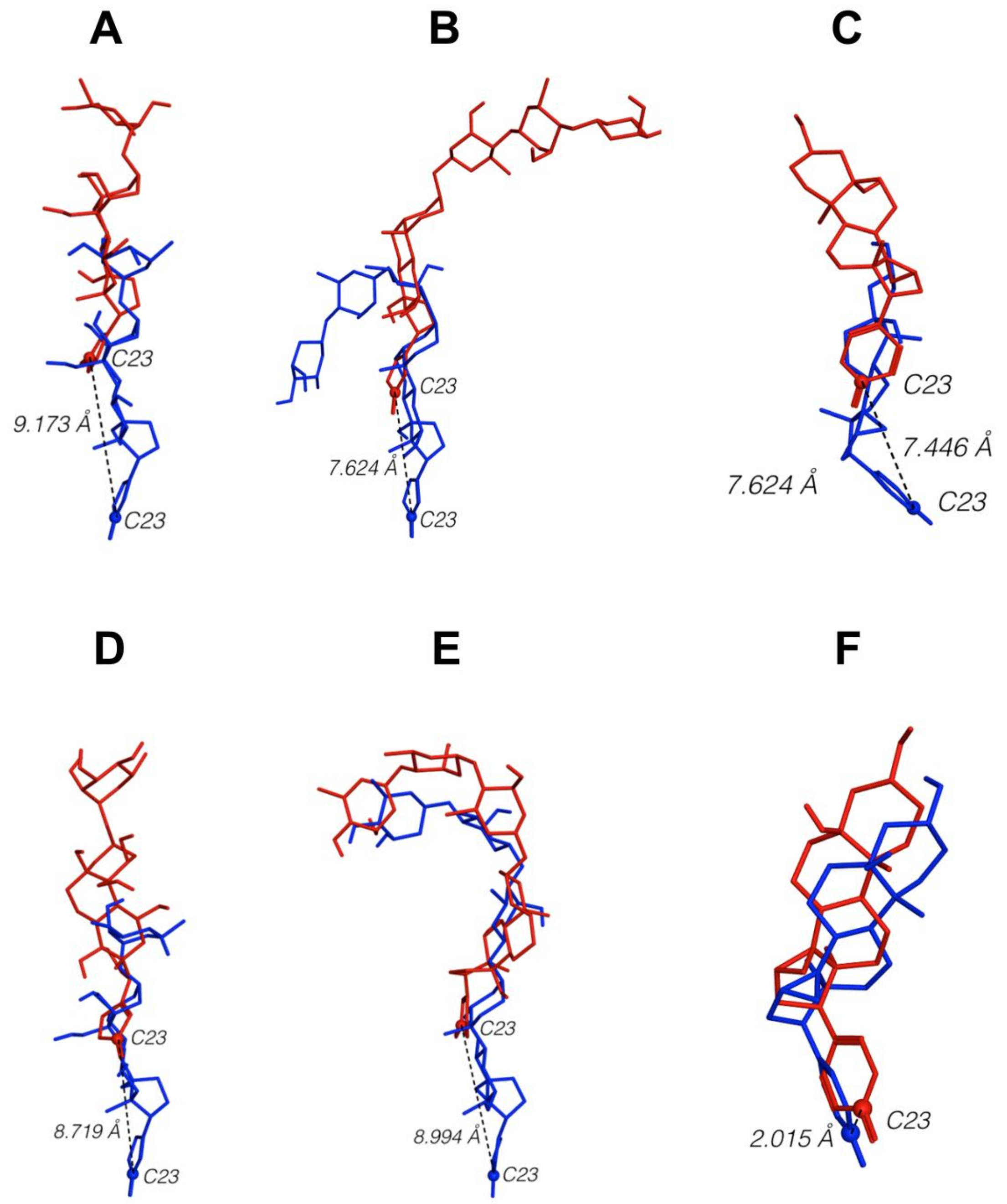

Figure S2. Superposition of cardiotonic steroids in the binding site of Na,K-ATPase. Difference in the deepening of the steroid nucleus of cardiotonic steroids between α1S-Na,K-ATPase and α1R-Na,K-ATPase: (**A,D**)—ouabain, (**B,E**)—digoxin, (**C,F**)—marinobufagenin. The distance was measured from C23 of the ligand docked to α1S-Na,K-ATPase to the ligand docked to α1R-NKA ((**A–C)** for the 4HYT; (**D–F)** for the 4RET model). Cardiotonic steroids from the α1S-Na,K-ATPase model colored in blue and from the α1R-Na,K-ATPase model colored in red.

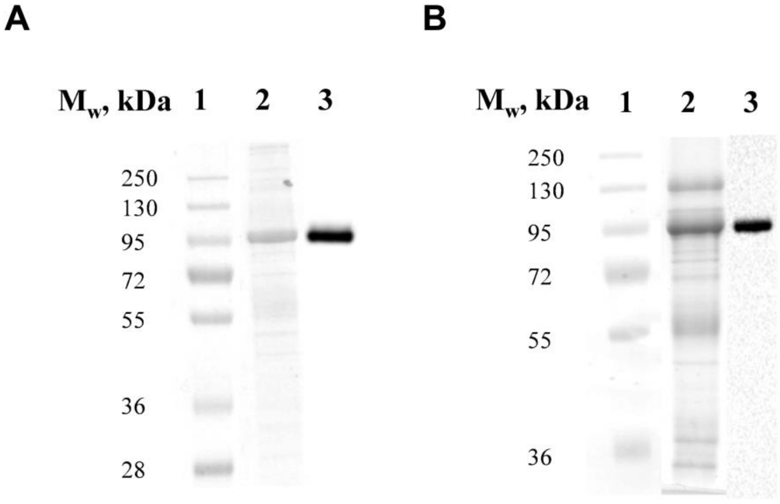

Figure S3. Analysis of purified Na,K-ATPase. Sodium dodecyl sulfate-polyacrylamide gel electrophoresis (lines 1–2) and Western blot analysis (line 3) of purified Na,K-ATPase from pig (**A**) and rat (**B**) kidneys. 1—molecular weight standards; 2,3—purified Na,K-ATPase.

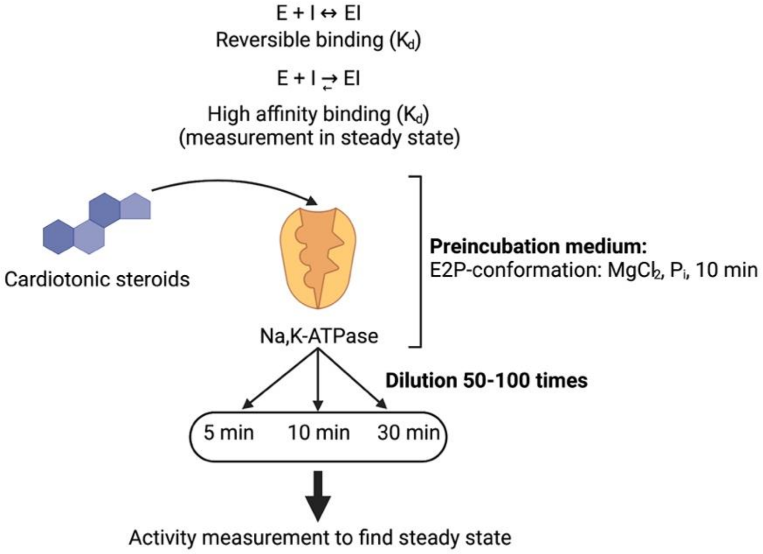

Figure S4. Scheme of the experiment to determine the type of inhibition. Na,K-ATPase in E2P-conformation was preincubated with different concentrations of cardiotonic steroids for different time intervals. Then aliquots of the preincubation medium were transferred into the incubation medium for the enzyme activity assay.
